# Flexural-Mode Piezoelectric Resonators: Structure, Performance, and Emerging Applications in Physical Sensing Technology, Micropower Systems, and Biomedicine

**DOI:** 10.3390/s24113625

**Published:** 2024-06-04

**Authors:** Xianfa Cai, Yiqin Wang, Yunqi Cao, Wenyu Yang, Tian Xia, Wei Li

**Affiliations:** 1 College of Integrated Circuit Science and Engineering, Nanjing University of Posts and Telecommunications, Nanjing 210046, China; xianfacai@njupt.edu.cn (X.C.); yiqinwang99@163.com (Y.W.); 2 State Key Laboratory of Industrial Control Technology, College of Control Science and Engineering, Zhejiang University, Hangzhou 310027, China; caoyunqi@zju.edu.cn; 3 School of Mechanical Science and Engineering, Huazhong University of Science and Technology, Wuhan 430074, China; mewyang@hust.edu.cn; 4 Department of Electrical and Biomedical Engineering, University of Vermont, Burlington, VT 05405, USA; txia@uvm.edu; 5 Department of Mechanical Engineering, University of Vermont, Burlington, VT 05405, USA

**Keywords:** piezoelectricity, resonator, flexural mode, micropower, sensor, biomedicine

## Abstract

Piezoelectric material-based devices have garnered considerable attention from scientists and engineers due to their unique physical characteristics, resulting in numerous intriguing and practical applications. Among these, flexural-mode piezoelectric resonators (FMPRs) are progressively gaining prominence due to their compact, precise, and efficient performance in diverse applications. FMPRs, resonators that utilize one- or two-dimensional piezoelectric materials as their resonant structure, vibrate in a flexural mode. The resonant properties of the resonator directly influence its performance, making in-depth research into the resonant characteristics of FMPRs practically significant for optimizing their design and enhancing their performance. With the swift advancement of micro-nano electronic technology, the application range of FMPRs continues to broaden. These resonators, representing a domain of piezoelectric material application in micro-nanoelectromechanical systems, have found extensive use in the field of physical sensing and are starting to be used in micropower systems and biomedicine. This paper reviews the structure, working principle, resonance characteristics, applications, and future prospects of FMPRs.

## 1. Introduction

Rapid advancements in microfabrication technology have led to the proposal of various micromechanical piezoelectric resonators, such as thin film bulk acoustic resonators (FBARs) [1,2], surface acoustic wave resonators (SAWRs) [3,4], and flexural mode resonators (FMRs) [5,6,7]. These resonators feature manufacturing processes that are compatible with mainstream integrated circuit technology. Among them, FMRs have gained extensive application across diverse fields due to their miniature, precise, and efficient performance [8,9,10,11]. They are extensively used in microelectronics [12,13], micromechanics [14,15], biomedicine [16,17], and chemical sensing [18,19], providing significant technical support for research and development in these areas. FMRs can be classified into groups of electrostatic excitation [20], electromagnetic excitation [21], piezoelectric excitation [7], and thermal excitation [22] based on the mode of excitation. Piezoelectric excitation, with its high energy density, ease of integration, frequency scaling, simple measurement, and low power consumption configuration, makes piezoelectric an attractive solution for various applications, such as sensors [19,23], actuators [24,25], energy harvesters [26,27], communication modules [28,29], etc. It has advantages of a large driving force, small parasitic capacitance, strong resistance to electromagnetic interference, and compact structure. However, it also has certain drawbacks, such as high environmental sensitivity and thermal stress issues associated with the piezoelectric thin film.

Piezoelectric excitation is extensively utilized in quartz resonators [30], zinc oxide (ZnO) resonators [31], and aluminum nitride (AlN) resonators [7]. Resonators made from other materials, such as silicon and silicon carbide, typically necessitate the bonding of piezoelectric material like ZnO, AlN, or lead zirconate titanate (PZT) [32,33]. For instance, a zinc oxide thin film sandwiched between two electrodes can be excited; the resulting voltage changes the film’s thickness and lateral dimensions, which can induce flexural mode vibration in the beam.

Flexural-mode piezoelectric resonators (FMPRs) are a novel resonator type that have garnered significant attention due to their unique characteristics and wide-ranging application potential [7,34,35]. The resonant frequency of FMPRs, like that of other FMRs, can be tuned using distinct active tuning methods such as external excitation [36] or local bias force [37]. Moreover, the high precision and efficiency of FMPRs have been instrumental in various precision equipment and high-performance systems. 

Resonators, particularly nano-resonators, possess a large surface-to-volume ratio, making them susceptible to environmental influences. Generally, nano-resonators must operate under ultra-low temperature and ultra-high vacuum conditions [38,39]. However, most studies are centered on biochemical reactions under atmospheric conditions, limiting the applicability of nano-resonators in biological reactions. Furthermore, existing ultra-low temperature and ultra-high vacuum systems, whether in terms of volume, weight, power, or cost, still constitute a major portion of the entire instrument [40]. Therefore, it is essential to examine the dissipation of resonators and optimize their design to ensure they can function under more challenging environmental conditions. 

**Figure 1:**
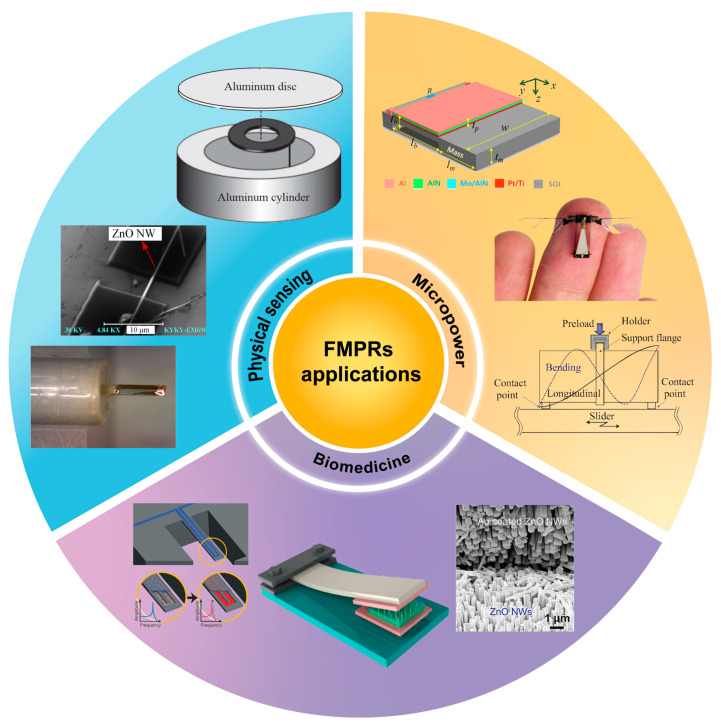
Applications of FMPRs.

While there have been numerous reviews on piezoelectric resonators [32,41,42], detailed reviews on FMPRs are lacking. This paper aims to fill that gap by reviewing the research progress on the resonance characteristics and applications of FMPRs. We first introduce the basic principle of FMPRs, followed by a detailed discussion on the theoretical and experimental research progress of resonance characteristics. Subsequently, we delve into the research progress in various application fields (Figure 1). Lastly, we discuss current challenges and future research directions. This paper is intended to serve as a comprehensive research reference for those interested in FMPRs, as well as to provide insights and references for the future development of FMPRs.

## 2. Basic Principles of FMPRs

### 2.1. Piezoelectric Effect and Piezoelectric Materials

The piezoelectric effect is a phenomenon whereby specific crystalline materials generate electric charges when subjected to mechanical stress. Conversely, these crystalline materials also undergo shape changes when an electric field is applied. This distinctive characteristic renders piezoelectric materials highly suitable for resonator manufacturing [32,33,43]. 

**Figure 2:**
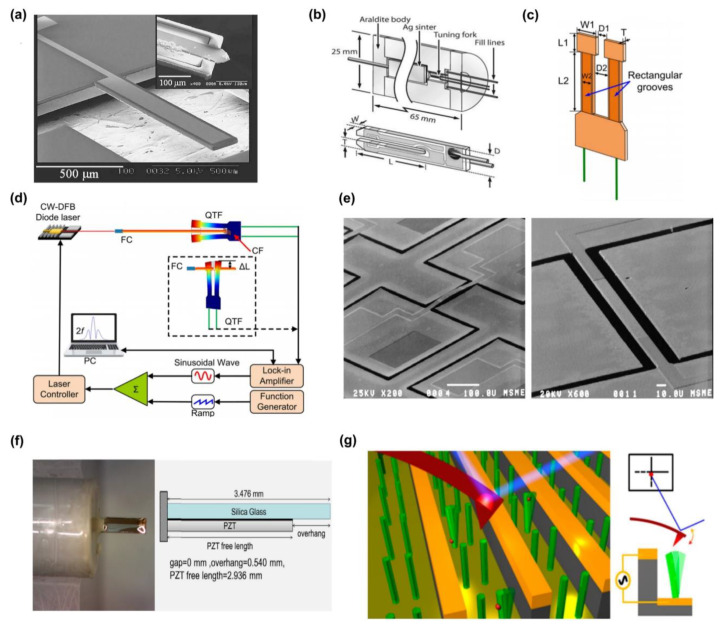
(**a**) SEM image of a typical quartz crystal cantilever. The inset shows a silicon cantilever tip mounted at the end of the quartz cantilever [52]. (**b**) Photoacoustic spectroscopy sensor based on a quartz tuning fork detector [53]. (**c**) IP-QEPAS sensor structure diagram and (**d**) system schematic diagram [51]. (**e**) Microscopic image of single-beam ZnO resonator [54]. (**f**) Structure of piezoelectric resonant sensor made of PZT material [55]. (**g**) Schematic diagram of vertical NW array vibration measured on a conductive substrate using AFM tip [56].

Commonly used piezoelectric materials in research and applications include Quartz, ZnO, AlN, sodium niobate, lithium niobate, and PZT [41,44,45]. These materials have attracted significant interest due to their inherent chemical stability, non-toxicity, cost effectiveness, and simplified manufacturing processes. Quartz, with its unique piezoelectric effect, is utilized in the manufacturing of acoustic devices, a notable application being the production of piezoelectric resonator devices. In contemporary communication realms, quartz crystal resonators serve as frequency control electronic components, offering frequency standards. They constitute the core components of oscillators and filters, stabilizing frequency, and enabling frequency selection and detection [46]. Furthermore, quartz crystal resonators are employed to manufacture corresponding sensors due to their sensitivity to pressure, mass, and acceleration [47]. The vibration modes in quartz crystals, encompassing flexural mode, extension mode, face shear mode, and thickness shear mode, are primarily dictated by the quartz crystal cut. Typically, XY and NT cut quartz crystals majorly vibrate in the flexural mode [48], as depicted in Figure 2a. Currently, the majority of mass-produced quartz crystal tuning fork resonators vibrate in the flexural mode. Their structure, as illustrated in Figure 2b, permits temperature and angular velocity detection by monitoring the tuning fork resonators’ vibration signals [49,50]. In 2020, Ma et al. [51] introduced an optical gas sensing technique based on in-plane quartz-enhanced photoacoustic spectroscopy (IP-QEPAS). The sensor’s structure and detection system are displayed in Figure 2c,d, respectively. By selecting water vapor as the target gas, the signals generated by the IP-QEPAS sensor were found to surpass those measured using traditional configuration structures by over 40 times. 

Porous micro-nano ZnO materials, owing to their exceptional gas-sensitive properties, have been widely utilized in resistive semiconductor gas sensors [57]. When applied to resonant microcantilever beams, this type of piezoelectric material can serve as a gas-sensitive sensor with heightened sensitivity. Figure 2e demonstrates a simple three-mask manufacturing process using a zinc oxide thin film, showcasing the ZnO dual-clamped piezoelectric beam resonator with a central frequency range of 158 kHz to 1.18 MHz [54]. However, since ZnO’s piezoelectric coefficient is significantly lower than that of PZT, another piezoelectric material, substituting ZnO with PZT, presents a simple and logical method to enhance the resonant micro-beam’s sensitivity. This strategy has been validated in research [33,58]. Rosario et al. [55] constructed a piezoelectric-excited millimeter-sized cantilever (PEMC) sensor by combining a 127 µm thick piezoelectric layer (PZT) with a 160 µm thick silicon base layer, as illustrated in Figure 2f. The study established that the fabricated PEMC sensor could measure gas density changes with an accuracy as low as 0.088 g/L, and a sensitivity equivalent to 0.049 g/(L·Hz). 

With the advancement in one-dimensional ZnO material fabrication technology, an increasing number of single-beam ZnO resonators and their corresponding arrays have been deployed. These resonators offer superior quality factors and frequency stability compared to their carbon nanotube counterparts [31]. Jiang et al. [56] employed self-assembled ZnO nanowire (NW) arrays to construct chip-sized vertically aligned NW resonator array devices using a straightforward one-step photolithography process, as illustrated in Figure 2g. They introduced a novel method for atomic displacement sensing via atomic force microscopy (AFM), capable of effectively identifying the resonance of 50 nm diameter NW resonators within an atmospheric environment. These resonators can operate in the linear induction voltage regime with an average quality factor of 1020 at 1 atm and room temperature. 

Concurrent with the swift progress of nanotechnology, new nanostructured materials possessing piezoelectric properties, including piezoelectric NWs, nanofilms, and nanosheets, are being continually developed. These materials significantly improve the resonant frequency, sensitivity, and precision of nano-piezoelectric resonators while reducing their power consumption [32].

### 2.2. Resonance Characteristics and Quality Factor

**Figure 3:**
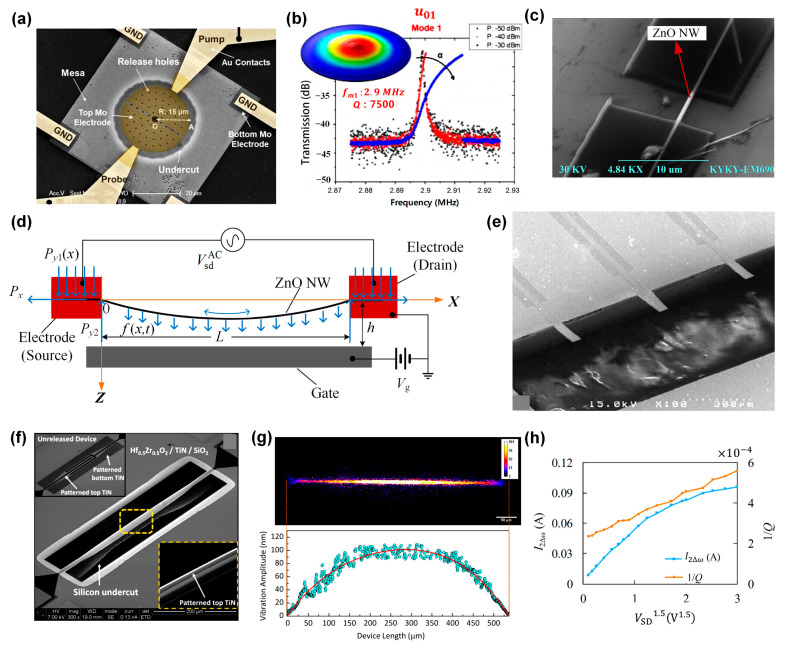
(**a**) SEM image of a stand-alone AlN-on-Si micromechanical resonator and (**b**) the flexural-mode characteristics of thin-film circular membrane resonator [59]. (**c**,**d**) The structure and mechanical model of the piezoelectrically excited ZnO NW, respectively [21]. (**e**) Resonator for monitoring the mass accumulation rate of single cells [67]. (**f**) Structure of the Hf_0_._5_Zr_0_._5_O_2_ thin film and (**g**) the contour map of the vibration amplitude of the entire device. The red curve is the fitting curve of the blue data points, showing the fitted mode-shape profile of the device [68]. (**h**) Effect of piezoelectric excitation voltage on output current and dissipation of the ZnO NWs resonator [21].

FMPRs function by exploiting the flexural mode vibration of piezoelectric materials. Flexural-mode vibration refers to the flexible oscillation of an object around an axis due to external forces. Typically, FMPRs operate under the first-order mode. Figure 3a,b illustrate a circular membrane resonator’s structure diagram coated with an aluminum nitride thin film on silicon and an example of its flexural vibration mode, respectively [59]. This type of vibration has the advantage of generating a larger displacement at a lower frequency and a smaller driving force. It largely depends on the piezoelectric coefficient *e*_31_ for inter-domain energy coupling [60]. This provides FMPRs with high sensitivity and low energy consumption, making them favorable for micro-nano-scale applications.

The resonator’s resonance characteristics directly influence its performance in practical applications. Hence, a thorough understanding of FMPRs’ resonance characteristics is crucial for optimizing their design and enhancing their performance. Generally, resonators with smaller size and lighter mass have a higher resonant frequency, thus higher sensitivity. Compared to micro-resonators, nano-resonators possess a smaller equivalent mass and higher resonant frequency, resulting in greater sensitivity [61,62]. They have significantly contributed to the measurement of minuscule masses [63], elucidating the dynamics of interactions and biochemical reactions between tiny masses [16,17], potential atomic or molecular scale dynamics detection [64,65], and even quantum mechanics analysis [38,66].

One-dimensional FMPRs possess a high resonant frequency and can produce a high signal-to-noise ratio output signal with minimal driving force. Their force sensitivity can reach 0.1–10 fN Hz^−1/2^, making them ideal for measuring the mass in the range of attograms (ag) to femtograms (fg). Cai et al. [21] employed a top-down processing method to fabricate ultra-high-frequency (UHF, 300 MHz–3 GHz) FMPRs based on defect-free single-crystal ZnO NWs. The ZnO NW resonator exhibits high sensitivity and low energy loss and can be detected in a room-temperature environment. The resonator’s structure and the mechanical model of the piezoelectrically excited ZnO NW are depicted in Figure 3c,d, respectively. Nano-beams with inverse piezoelectric properties (such as ZnO NWs) generate stretch movements at the same frequency as the excitation signal when exposed to alternating voltage. Consequently, under the impact of bias voltage pre-tightening, flexural-mode vibration is produced. A driving force of 33 × 10^−12^ N can generate a resonant beam amplitude of 0.8 nm and an output current signal of 0.1416 A, with force sensitivity reaching 1.23 × 10^−15^ N·Hz^−1/2^. 

However, due to the considerable surface area of nano-beams, they are susceptible to external environmental factors and typically require ultra-low temperature conditions for optimal performance [38,39]. Therefore, in demanding work environments where high reliability and a larger surface area of the resonant beam are required, such as in the application of resonators for the detection of biological particle reactions, the utility of micro-beam resonators is unparalleled. Lee et al. [67] constructed a cantilevered nano-resonator, utilizing PZT as a composite layer of the excitation thin film on silicon nitride (SiN), as depicted in Figure 3e. This approach enables the interaction between antigen (Ag) and antibody (Ab) molecules to induce changes in resonance frequency, facilitating label-free detection of prostate-specific antigen (PSA) under regulated temperature and humidity conditions. It boasts a detection sensitivity as low as 10 pg/mL and can also be employed to investigate the structure of deoxyribonucleic acid (DNA) ligand complexes, as well as internal changes in double-stranded DNA under differential action. Ghatge et al. [68] introduced an integrated nanomechanical resonator, founded on the atomic engineering of a ferroelectric hafnium zirconium oxide (Hf_0_._5_Zr_0_._5_O_2_) thin film as portrayed in Figure 3f. By leveraging the robust electrostriction effect present in the Hf_0_._5_Zr_0_._5_O_2_ ferroelectric material, they excited a 30 nm thick nanomechanical resonator to a 195 kHz flexural resonance, yielding a vibration amplitude of 100 nm, as illustrated in Figure 3g. This resonator exhibited quality factors (*Qs*) of 15 and 3300 under atmospheric and 10^−7^ torr environmental pressures, respectively. Its atomic-level thickness, fully integrated operation, high *Q* value, and robust features show promising potential for applications in information processing and sensing. 

To attain room-temperature detection with FMPRs, it is imperative to minimize the energy dissipation induced by the resonant structure’s vibration and enhance the sensor’s quality factor. This entails mitigating various forms of energy dissipation that result from the coupling of the resonant structure vibration with its surrounding environment. By reducing energy dissipation, the resonator’s quality factor can be improved. Resonators of this type exhibit excellent frequency selectivity, high sensitivity, low energy loss, and ease of detection. They also enable isotope or chemical identification, making the study of the resonator’s loss mechanism highly significant.

Resonant structure vibration’s coupling with the surrounding environment typically results in diverse types of energy dissipation governed by numerous internal and external mechanisms [35,69]. Common external energy dissipation examples include contact loss, clamping loss, surface modification loss, thermoelastic loss, air friction, and miscellaneous dissipation caused by various excitation methods [70,71]. Clamping loss can be reduced by increasing the clamping thickness of the resonator’s fixed end. Surface modification loss and air friction loss can be mitigated using surface treatment methods to decrease resonator surface adsorption during processing, limit the resonator’s exposure time in the atmosphere, and conduct storage and experimentation in a high-vacuum environment. However, thermoelastic loss is an inherent resonator loss that is challenging to eliminate through design optimization [70,72]. Some energy dissipation forms can be attributed to the material’s inherent characteristics. For instance, energy dissipation due to the rough surface of NWs and some internal defects such as atomic vacancies (point defects) exemplify this inherent energy dissipation. 

If the coupling effect of factors causing dissipation is disregarded, the calculation formula for the quality factor of FMPRs is expressed as follows [73]:

(1) $$\frac{1}{Q}=\frac{1}{Q_{M}}+\frac{1}{Q_{V}}=\frac{1}{mf_{0}}\left(\beta_{M}+\beta_{V}\right)$$

where the local quality factor and damping constant related to mechanical dissipation are denoted as $Q_{M}$ and $\beta_{M}$, respectively, while $Q_{V}$ and $\beta_{V}$ symbolize the local quality factor and damping constant related to piezoelectric dissipation. 

Using a one-dimensional piezoelectric resonator with two fixed ends as a reference, the piezoelectric damping constant can be expressed as [21]

(2) $$\beta_{V}=\gamma {\left(V_{\mathrm{sd}}^{\mathrm{AC}}\right)}^{3/2}$$

where *γ* represents a constant estimated from experimental data, and $V_{\mathrm{sd}}^{\mathrm{AC}}$ is the alternating excitation voltage applied across ZnO NW.

From Equation (2), it is evident that a linear relationship exists between 1/*Q* and ${\left(V_{\mathrm{sd}}^{\mathrm{AC}}\right)}^{3/2}$ when piezoelectric dissipation dominates. Typically, for resonators with efficient clamping at the fixed end and functioning in near-vacuum conditions, piezoelectric dissipation is the primary form of loss in FMPRs [21]. As illustrated in Figure 3h, piezoelectric damping is the dominant factor, with clamping dissipation still playing a significant role in overall piezoelectric dissipation. Therefore, it can be inferred that a linear relationship exists between 1/*Q* and ${\left(V_{\mathrm{sd}}^{\mathrm{AC}}\right)}^{3/2}$ to a certain degree. The intercept of the linear fitting line of 1/*Q* on the *y*-axis represents the total dissipation excluding the effect of piezoelectric damping. 

Several methods have been proposed by researchers to enhance the quality factor of the resonator, including improving the precision of the detection circuit [74], operating in ultra-low-temperature high-vacuum conditions [38], implementing feedback circuits [75], and utilizing single-crystal defect-free materials [21]. Numerous studies indicate that the material density at the clamping end could potentially influence the strain of the resonator, which is coated on both ends of the NWs using evaporation plating [21,71,76]. Larger strain is associated with greater clamping loss [21]. Hence, for resonators with small aspect ratios, piezoelectric excitation can effectively decrease vibration dissipation and enhance the quality factor of the resonator compared to other excitation methods. For resonators with large aspect ratios, clamping loss ceases to be the primary loss, and losses caused by the excitation method become dominant. Table 1 provides an extensive review of the resonance characteristics of FMPRs made of varying materials. It is evident that the primary types of FMPRs include tuning forks, cantilever beams, two-end-fixed beams, and circular diaphragms. Typically, nanoscale FMPRs require stringent operating conditions, needing to function in nearly vacuum environments, with some even necessitating ultra-low temperatures. The resonance frequency of FMPRs generally ranges from several thousand Hz to a few hundred MHz.

There exist inherent challenges in utilizing piezoelectric materials in resonators. The primary issue arises from the fact that piezoelectric materials are highly sensitive to external environments such as humidity and temperature [8,77]. Furthermore, the application of thin films on the resonator results in a decrease in the quality factor due to the disparity in thermal expansion.

## 3. Applications of FMPRs

Conventional piezoelectric resonators predominantly employ longitudinal or radial vibrations. For instance, disk-shaped piezoelectric oscillators can be utilized in acoustic energy collection, piezoelectric transformers, accelerometers, and so forth [59]. Nonetheless, several significant issues hinder the wide-scale application of piezoelectric oscillators in power electronic systems [78,79]. First, the piezoelectric driving force results in an unstable oscillator support point and reduced precision, potentially leading to device damage when the driving force is excessive. Second, the inherent dielectric loss of the oscillator is relatively high, negatively impacting the quality factor. Lastly, the vibration mode leads to insufficient actual power transmission capacity. 

FMPRs can mitigate such issues as displacement reduction due to vibration, electrode lead solder joint loosening, and piezoelectric sheet failure due to cracking. This enhances the device’s reliability while decreasing dielectric loss and significantly improving power transmission capacity. Given their high integration and superior resonance characteristics, FMPRs hold considerable potential for application in physical sensing technology, micropower systems, and biomedicine.

### 3.1. Applications in Physical Sensing

The use of FMPRs in physical sensors has garnered significant attention in recent years. Their distinctive resonance characteristics make them particularly adept at accurately measuring physical variables, such as pressure, temperature, humidity, acceleration, ultrasonic, concentration, and force [80,81,82]. Through the monitoring of subtle changes in resonant frequency, precise measurements of these variables can be achieved. 

**Figure 4:**
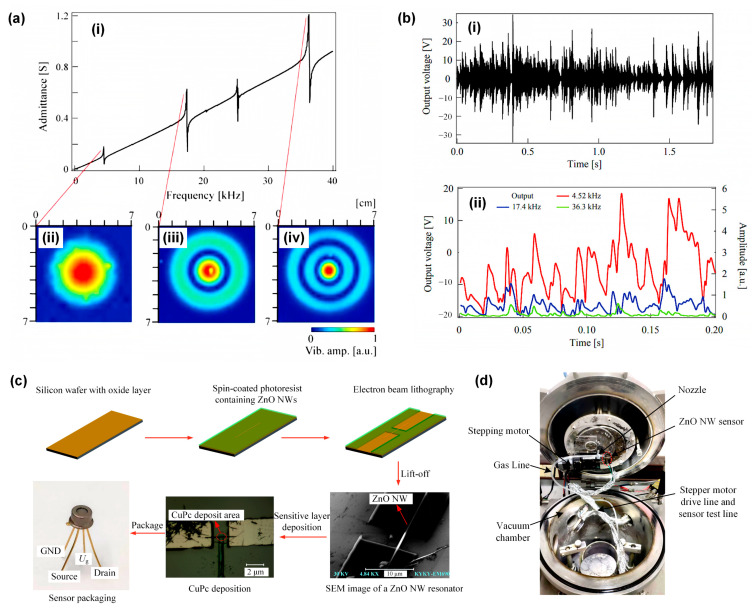
(**a**) The axial symmetrical flexural-mode vibration of the disc-shaped piezoelectric sensor, where (**i**) frequency characteristics of the electric admittance of the sensor and vibrational distributions on the sensor surface at resonance frequencies of (**ii**) 4.52, (**iii**) 17.4, and (**iv**) 36.3 kHz are depicted [84]. (**b**) Monitoring output signal for the riverbed load of gravel, where (**i**) output voltage waveform of sensor generated upon sequential impacts with crushed stones and (**ii**) changes in spectrum amplitudes at resonance frequencies of 4.52, 17.4, and 36.3 kHz are depicted [84]. (**c**) The top-down method of making ZnO NW resonant gas sensors and (**d**) their detection devices [76].

FMPRs are highly effective for sensing physical quantities such as pressure, temperature, and humidity. They exhibit wide-band characteristics, enabling measurements and monitoring across diverse frequency ranges [83]. Notably, microscale resonators produce stable and reliable output signals, are minimally influenced by environmental factors, boast an extended service life and durability, and are thus well suited for long-term monitoring and applications. These devices excel in pressure–frequency characteristics, enabling high-resolution pressure measurements. Furthermore, the flexural mode of the resonator facilitates a larger frequency shift due to pressure, thereby improving the sensor’s accuracy. Sakata et al. [84] developed a passive piezoelectric sensor for measuring particle size distribution and performed a continuous measurement of continuous impact. The sensor, made of an aluminum disc and an annular piezoelectric sensor, is ideal for real-time monitoring of gravel load on river beds. When gravel strikes the sensor board surface, the sensor enters a flexural mode and generates electricity via the piezoelectric effect. Its resonance frequencies, corresponding to the axisymmetric flexural-mode vibration modes, are depicted in Figure 4a. The overlapping output signals of the sensor due to the sequential gravel impact can estimate the grain size distribution of gravel through time-frequency analysis, as illustrated in Figure 4b.

Piezoelectrically excited ZnO resonant gas sensor platforms offer several advantages, such as compact size, low power consumption, sensitive response, and convenient array implementation. By coating sensitive materials at specific locations on the resonant beam, a range of sensors can be produced, particularly for gas detection. For the resonant beam, changes in effective mass are directly related to the concentration of particular chemicals in the environment. However, in actual operation, various factors may influence the resonance frequency of the device, including temperature and humidity changes, and the potential binding of other molecules to the resonant beam. As such, to minimize interference, high-precision detection is typically conducted in a low-vacuum environment. Cai et al. [76] created a ZnO NW resonant gas sensor using a top-down approach, as shown in the production process in Figure 4c. Utilizing a very high-frequency detection technology based on a phase-locked amplifier, they discovered that piezoelectrically excited ZnO NW can operate at a resonant frequency of 417.35 MHz and a quality factor of 3010 under room-temperature conditions. To enhance the test’s accuracy, the sensor’s sensitive area was positioned at the midpoint of the resonant beam during production, harmonizing the step difference of the single-molecule adsorption response. The detection device is depicted in Figure 4d. The resonator’s mass sensitivity reached −8.1 Hz/zg, the resolution was 192 zg, and it demonstrated the capacity to detect biochemical reactions of biological particles such as viruses, DNA, and protein molecules.

FMPRs exhibit exceptional sensing characteristics for physical measurements, underscoring their significant potential in the realm of structural health monitoring. Structural health monitoring, a real-time technique for monitoring and evaluating the status of structures such as buildings, bridges, and aircraft, can greatly benefit from FMPRs. These devices, renowned for their high sensitivity and broad bandwidth, are adept at capturing minute vibration changes. This makes them suitable for structural vibration monitoring and damage diagnosis. Their high sensitivity and wide bandwidth allow for them to detect small vibrational changes within structures accurately, facilitating real-time monitoring and early warning of the structure’s health status [85,86]. 

Among various FMPRs, the thin-film-on-silicon (TPoS) resonator stands out due to its small size, low power consumption, high electromechanical conversion efficiency, and the ability to realize single-chip multi-devices, making it a popular choice in the sensing field. Traditional miniature sensors using TPoS resonators often employ wired sensing methods, which limits portability and application scenarios. In recent years, however, there has been growing interest in wireless sensing technology for TPoS resonant sensors [87,88,89]. This technology, which combines the TPoS resonator with a near-field coupling coil to form a wireless sensor, shows great promise for a wide range of applications. However, it does come with challenges, such as weak sensing signals and shorter sensing distances.

### 3.2. Applications in Micropower Systems

The piezoelectric effect, a primary impact between the mechanical and electrical parameters of solid dielectrics, can only occur in dielectrics devoid of central symmetry. This effect underlies the working principle of piezoelectric drive, which hinges on the crystal’s inverse piezoelectric characteristic. Essentially, the inverse piezoelectric effect involves the conversion of electrical energy into mechanical energy. Piezoelectric micro-actuators, known for their high displacement resolution, control accuracy, quick response, substantial driving force, and low driving power, make use of this inherent mechanical–electrical coupling effect [11,90]. As such, these materials have proven widely applicable in engineering and have been extensively utilized in aerospace, precision machinery, and energy collection sectors [91,92]. 

**Figure 5:**
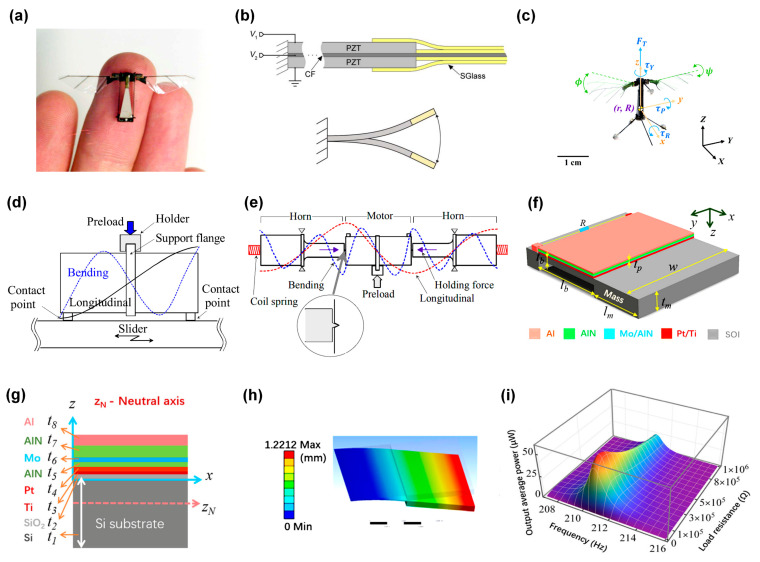
(**a**) RoboBee aircraft overall structure, (**b**) flapping drive structure [94], and (**c**) RoboBee under harmonic sine drive [96]. (**d**) A bolt-clamped Langevin-type longitudinal and bending hybrid transducer (BLT), (**e**) the holding mechanism of its step angle resonant support mechanism [99]. (**f**) Schematic diagram of the cantilever aluminum nitride (AlN) vibration energy collector structure, (**g**) its sectional view, (**h**) its first-order mode, and (**i**) the output average powers with different load resistances and different excitation frequencies. The darker the red color, the greater the output average energy [101].

Smart structures constructed using piezoelectric materials are gaining momentum. These structures, besides having a self-sustaining capacity, also possess self-diagnosis, self-adaptation, and self-repair functions. Thus, they assume a pivotal position in future aircraft design. FMPRs are extensively used in the flapping drive of flapping aircraft. Piezoelectric excitation, with its simple structure and driver control ease, is superior to electromagnetic and electrostatic excitation [93]. A notable application is Harvard University’s “RoboBee”, which has achieved significant advancements in flapping aircraft manufacturing. In 2007, Professor Wood from Harvard University’s Microrobotics Lab successfully developed the world’s first piezoelectrically excited imitation insect flapping aircraft capable of overcoming its own gravity and taking off vertically along a vertical guide rail [94]. The overall aircraft structure and the flapping wings’ driving structure are depicted in Figure 5a,b, respectively. Subsequent step-by-step optimization of the aircraft introduced a new four-flapping flight structure, increasing its lift and controllability [95], and implemented a harmonic sine control model to augment yaw and torque control [96], as shown in Figure 5c.

FMPRs also find application in micro-displacement systems. With the rapid advancement of ultra-precision machining and micro-nano manufacturing, there is an increasing demand for journey, speed, and accuracy in drive and positioning systems. This necessitates actuators with extensive journey, high speed, and ultra-precision driving capabilities. However, the positioning accuracy of electromagnetic motors generally only reaches the sub-micrometer level. Although resonant piezoelectric actuators boast fast drive, large thrust, and a significant stroke, they also suffer from issues such as limited drive displacement, constant response time or vibration frequency, and positioning accuracy remaining at the micron or sub-micron level. This somewhat restricts their practical application and does not align with the commercialization trend of MEMS products [97,98]. To address these issues with piezoelectric actuators, comprehensive and mature research has been conducted in Japan. Scholars from the Precision and Intelligence Laboratory of Tokyo Institute of Technology, such as Yun et al. [99], have proposed a fixed system using resonance for longitudinal bending piezoelectric actuators. The basic motor configuration and the holding mechanism using resonance are illustrated in Figure 5d,e, respectively. This system allows for the ultrasonic motor to drive a 10 kg platform at a speed of 200 mm/s, with a platform positioning accuracy of 50 nm. Additionally, other researchers [97,98,100] have conducted extensive research on resonant piezoelectric actuators, yielding numerous valuable research findings.

FMPRs, renowned for their high conversion efficiency and stable performance, are extensively applied in the domain of environmental energy collection. Here, they primarily function to harness mechanical energy from the natural environment and transmogrify it into electrical energy, thereby energizing micromachines [102,103]. This process is typically employed to collect energy generated from renewable sources such as wind and water. He et al. [101] conceptualized a cantilever-type aluminum nitride (AlN) vibration energy collector. Its structural design, sectional view, and the first-order mode are depicted in Figure 5f–h, respectively. Figure 5i illustrates the output average power under varying load resistances and excitation frequencies. Upon evaluation, it was observed that the maximum output average power and output average power density reached peaks of 56.4 μW and 854.55 μW/cm^3^/g^2^, respectively. This indicates that the device holds promising potential for wide-ranging applications within the energy collection sphere. 

### 3.3. Applications in Biomedicine

FMPRs are highly sensitive, with a broad frequency response range and stability. Their wide-ranging applications in biomedical sensing, such as biomolecular detection and body fluid analysis render them indispensable tools in medical equipment, diagnosis, and health monitoring. Owing to their rapid response speed, FMPRs can measure pressure, force, and motion of organisms in real time, making them suitable for applications like blood pressure monitoring and bone mechanics analysis. Additionally, FMPRs can function as nano-power sources, providing power and control for implantable medical devices.

**Figure 6:**
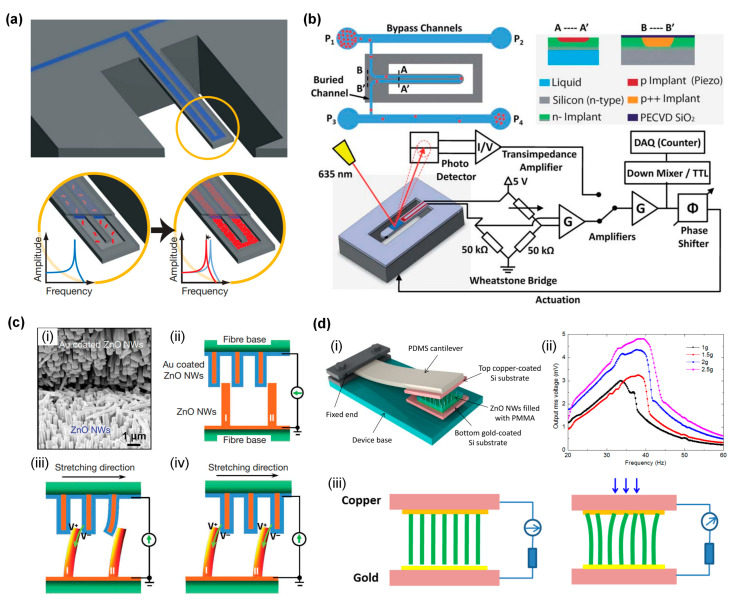
(**a**) Cantilever beam with micro-nano channels integrated into the mechanical resonator. The red curve represents the changed spectrogram [109]. (**b**) Micro-nano channel resonator piezoresistive and laser detection system [105]. (**c**) Low-frequency fiber nanogenerator driven by external pulling force. (**i**) Scanning electron microscope image of the “tooth-to-tooth” interface between two nanowire-covered fibers. (**ii**) Schematic diagram of the “tooth-to-tooth” contact between two nanowire-covered fibers. (**iii**) Under the traction of external force, the piezoelectric potential generated on NW I and NW II by the top fiber. (**iv**) Once the two NWs finally come in contact, it leads to further output current [122]. (**d**) A low-frequency ZnO NW energy collector. (**i**) Energy collector schematic. (**ii**) Device frequency domain output voltage at accelerations of 1 g, 1.5 g, 2 g, 2.5 g. (**iii**) The working principle of the piezoelectric discharge energy produced by ZnO NWs [123].

FMPRs can serve as highly sensitive biosensors capable of detecting minute changes in the mass of biomolecules [104,105,106]. The resonance frequency of the resonator changes when molecules are adsorbed on its surface. By attaching specific biomolecules or antibodies onto their surface, FMPRs can identify the presence of certain molecules or antigens in biological samples. Compared with biosensors not utilizing the principle of sensitive material vibration [107,108], these sensors exhibit superior sensitivity and selectivity, making them useful for disease diagnosis and drug discovery. In cases where chemicals need to react in liquid, mechanical resonators can integrate micro-nano channels, thus confining the fluid to internal channels to circumvent the influence of additional damping effects [109], as depicted in Figure 6a. Micro-nano channel mechanical resonators can also be used for sorting, capturing, and manipulating microparticles like cells [110,111,112], and characterizing fluid density [113], fluid viscosity [114], fluid phase change [115], particle position [116], and so on. Precise vibration control can facilitate cell separation, reagent mixing, or enhancement of chemical reactions in micro-scale channels. These resonators can be incorporated into compact diagnostic tools for real-time detection, improving the accessibility and convenience of healthcare. Lee et al. [104,105] embedded nano-scale channels (3.0 µm × 0.7 µm) in the beam structure, employing piezoresistive and laser detection as illustrated in Figure 6b, and managed to improve the mass resolution to 27 attograms (ag). Building on the work of Lee et al., Olcum et al. [106] further enhanced the mass resolution by reducing the resonator size and improving the excitation form, among others. They developed four sizes of micro-nano channel mechanical resonators, the smallest being a resonator with a 22.5 µm × 7.5 µm × 1.0 µm micro cantilever beam, embedded with a 1.0 µm × 0.4 µm nano channel in the beam, and a resonant frequency as high as 2.87 MHz. They also replaced electrostatic excitation with the piezoelectric excitation method, which increased the amplitude of the resonator and effectively reduced frequency noise. By comparing the mass distribution of exosomes produced by different cell types and characterizing the yield of self-assembled DNA nanoparticle structures, they demonstrated this capability’s potential, raising the mass resolution of the resonator to 0.85 ag.

The excitation method of the micro-nano channel mechanical resonator significantly influences system stability. The two most prevalent excitation methods are piezoelectric and electrostatic excitation. With electrostatic excitation, an overly large applied voltage can cause the beam’s elastic restoring force to fail to counterbalance the electrostatic force, inducing instability in the resonator. Hence, under electrostatic excitation, the micro-nano channel mechanical resonator can display two forms of instability: pull-in instability and flow-induced instability. Piezoelectric excitation also impacts the flow stability of the resonator. To enhance system stability, Abbasnejad et al. [117] accounted for the influence of the piezoelectric layer on the beam’s top and bottom surfaces, deduced the fluid–solid coupling dynamics model of the flow-through micro-beam, and significantly diminished the effect of flow speed on vibration frequency through the voltage difference between the piezoelectric layers, thus improving the system’s stability range. 

FMPRs have potential applications in implantable medical devices. They could offer power and control for drug delivery systems, sensors, or micro-scale actuators for treatment and diagnostic purposes. By providing precise control of mechanical vibrations on the micro-nano scale, piezoelectric micro-nano resonators open new possibilities for biomedicine. As these resonators’ potential in various aspects of healthcare and medical technology is explored, their applications continually evolve. For some biomedical nano-devices implanted in the body, traditional batteries are unsuitable power sources due to their large size, need for regular replacement or charging, and toxicity. Therefore, developing a nano power source that can self-power by harnessing human energy has become an ideal solution. Wang et al. proposed an innovative method to convert human mechanical energy (such as body movement, muscle stretch) into electrical energy using ZnO NW arrays, thus developing a DC nanogenerator, as depicted in Figure 6c. Consequently, many research groups worldwide have dedicated themselves to researching ZnO nanogenerators, using various methods to overcome the small output voltage bottleneck that limits practical applications [118,119]. They have prepared different types of nanogenerators on various substrates, including gallium nitride (GaN), silicon (Si), indium tin oxide (ITO), and other hard substrates [120], as well as flexible nanogenerators based on fibers, polyimide, polydimethylsiloxane, polystyrene, and other substrates [121], as depicted in Figure 6d.

With their high energy density, easy integration, good stability, and high sensitivity, FMPRs have considerable potential in several future fields: (1) In medical equipment and diagnosis, FMPRs can measure human biomechanical signals, such as electrocardiograms, electromyograms, electroencephalograms, blood pressure monitoring, and bone mechanics. (2) As drivers, FMPRs can control drug delivery systems by producing precise mechanical vibrations to regulate the release of drugs from micro/nano particles, thereby enabling targeted and controllable drug delivery, which is especially crucial in treating diseases like cancer. (3) In tissue engineering, these resonators can be used to study and manipulate the behavior of neurons. The precise mechanical vibrations they produce can stimulate or inhibit neural activity, leading to advances in neuroscience research and potential treatments for neurological diseases. These resonators can be used for intracellular research, introducing mechanical disturbances into individual cells to understand their response to mechanical signals. This is extremely important for studying cell biology and tissue mechanics.

## 4. Summary and Future Prospects

This review highlights the significant advancements in the study of FMPRs in recent years, primarily due to their compact, precise, and efficient performance. Their unique resonance characteristics and diverse applications have stimulated interest in various fields, including physics, engineering, and biomedical sciences. The review discusses the use of different piezoelectric materials in the construction of these resonators, emphasizing the importance of resonance characteristics in optimizing design and enhancing performance. Furthermore, it underscores the continuous expansion of application areas for these resonators, facilitated by the swift development of micro-nano electronics. FMPRs have found extensive applications in physical sensing, micropower systems, and biomedicine, providing vital technical support in these fields.

However, despite substantial progress, several challenges still need addressing. A primary challenge is the sensitivity of FMPRs to environmental conditions. The high surface-to-volume ratio of these resonators makes them highly susceptible to external conditions, and some require operation under extreme circumstances such as ultra-low temperatures and ultra-high vacuum. Hence, to ensure the stable operation of FMPRs, it is crucial to minimize their energy dissipation by optimizing the structural design, altering the excitation mode, and modifying the working environment. Ongoing research aims to develop strategies to reduce these losses and enhance the quality factor of the resonators. An additional challenge is the environmental sensitivity of the piezoelectric thin films used in the resonators. These materials’ sensitivity to humidity, temperature, and other external environmental conditions presents a challenge to their application in resonators [124].

Despite these challenges, the future of FMPRs remains promising. They have demonstrated potential across a broad spectrum of applications, from physical sensing technology and micropower systems to biomedical applications. In physical sensing technology, the resonators can deliver accurate measurements of physical variables such as pressure, temperature, humidity, acceleration, concentration, and force. In micropower systems, the resonators can harvest environmental energy to power micromechanical devices. In the biomedical field, the resonators can function as highly sensitive biosensors to detect changes in biomolecular mass and power implantable medical devices. However, further research and development are required to surmount the current challenges and fully unlock the potential of these resonators. Future research could focus on improving the resonators’ design, developing new materials for their construction, and exploring new application areas. As the field evolves, FMPRs will undoubtedly continue to play an increasingly vital role in science and technology.

## Figures and Tables

**Table 1:** Resonance characteristics of FMPRs made of different materials.

Reference	Type	Materials/ Technology	Temperature, Pressure	Resonant Frequency	Quality Factor	Application, Resolution
[53]	Tuning fork	Quartz	5 mK–1 K, zero—25 bar	32 kHz	10^6^	Detecting turbulence resistance, -
[51]	Tuning fork	Quartz	Room temperature, atmospheric pressure	9.38 kHz	8850	Gas sensor, -
[54]	Doubly clamped	ZnO on SiO_2_	Room temperature, atmospheric pressure	158–1180 kHz	930–3700	Electromechanical filters, -
[55]	Cantilever	PZT on silica glass	Room temperature, atmospheric pressure	6 kHz	43	Gas sensor, 0.049 g/(l Hz)
[56]	Cantilever	ZnO	Room temperature, 1 atm	9.98 MHz	1020	Mass selecting sifter, 10^−20^ kg
[21]	Doubly clamped	ZnO	Room temperature, 10^–4^ mbar	600 MHz	2246	Force sensor, 1.23 fN Hz^−1/2^
[31]	Doubly clamped	ZnO	Room temperature, 10^–4^ mbar	417 MHz	3010	Mass detection, 8.1 Hz/zg
[59]	Circular film	AlN	Room temperature, atmospheric pressure	2.9 kHz	7500	-, -

## Data Availability

The research data are available upon reasonable request.
